# Fault-Tolerant Control for Active Suspension Systems Based on Fault Estimation and Compensation

**DOI:** 10.3390/s26134226

**Published:** 2026-07-03

**Authors:** Yuanchun Ding, Guanglong Li, Falu Weng, Lumin Tan

**Affiliations:** 1 Ganzhou Key Laboratory of Industrial Safety and Emergency Technology, Jiangxi Provincial Key Laboratory of Safety and Efficient Mining of Rare Metal Resource, Jiangxi University of Science and Technology, Ganzhou 341000, China; dyc@mail.ustc.edu.cn; 2 School of Electrical Engineering and Automation, Jiangxi University of Science and Technology, Ganzhou 341000, China; 10932014@zju.edu.cn

**Keywords:** active suspension, fault-tolerant control, adaptive observer, fault diagnosis

## Abstract

This paper investigates fault-tolerant control for active suspension systems with some actuator faults. The objective is to design a fault-tolerant controller such that the closed-loop systems are stable while having a prescribed level of disturbance attenuation performance. First, by introducing an output vector to describe the actuator faults, a quarter-car active suspension model including actuator faults is obtained. Second, based on the linear quadratic optimal control technique and linear matrix inequality theory, the optimal feedback controller is obtained such that the closed-loop system achieves desirable disturbance attenuation performance. Moreover, the fault estimation and compensation methods are also discussed based on matrix techniques and mathematical operations. The proposed method estimates online both the system’s critical state variables and the actual fault force of the actuator, which demonstrates strong practicality. In the end, simulation results demonstrate that the obtained observer can achieve high-precision estimation of state variables and fault forces. Moreover, when the suspension system is controlled by the obtained fault-tolerant controller, the root mean square values of body vertical acceleration and suspension dynamic travel are decreased by 14.59% and 16.98% respectively, compared with the controller without fault consideration. The effectiveness of the proposed method is verified.

## 1. Introduction

The suspension system serves as a critical component connecting the vehicle body to the wheels, and its performance directly influences both ride comfort and driving safety. Active suspension systems employ actuators to deliver primary forces in real time, thereby overcoming the performance limitations inherent in traditional passive suspensions. This approach provides an effective means of simultaneously enhancing both ride smoothness and handling stability, establishing active suspension as one of the key research areas within the field of automotive engineering [1]. The vehicle suspension system transmits forces between the wheels and the body while suppressing road vibrations, thereby ensuring ride comfort and driving stability [2]. Suspension control methods primarily encompass passive control, semi-active control, and active control [3].

The application of advanced control theory to active suspension systems is widely recognized as an effective approach to enhancing suspension performance. Active suspension control methods attenuate vehicle vibrations by generating counteracting forces through actuator outputs, thereby achieving active compensation for road surface excitation and effectively suppressing body vibrations [4]. Active suspension systems must balance ride comfort, handling performance and safety, yet these performance metrics inherently conflict with one another. Current active suspension research primarily focuses on control strategy development, with representative algorithms including Linear Quadratic Regulator (LQR) optimal control [5], robust H∞ control [6], sliding-mode variable structure control [7], and model predictive control [8]. Among numerous control strategies, LQR optimal control has gained widespread application in active suspension control [9] due to its ability to seek optimal solutions under clearly defined performance metrics.

Active suspension systems have attracted extensive attention due to their ability to improve both ride comfort and handling stability under various road conditions. In recent years, fault-tolerant control (FTC) for active suspension systems has become an important research topic, as actuator faults can severely degrade system performance and even cause safety hazards.

Recent studies have explored different FTC strategies for suspension systems. To handle suspension nonlinearity and reduce network transmission burden, a hybrid event-triggered T-S fuzzy FTC framework was constructed, yet it cannot separately quantify real actuator fault force [10]. Ni et al. [11] provided a comprehensive review of fault-tolerant control methods from road vehicles to maglev trains, highlighting the challenges in practical implementation. Zhao and Gu [12] proposed a fractional-order sliding-mode controller based on RBF neural networks for nonlinear active suspensions, which effectively improves control performance under faults. Lopez et al. [13] implemented fault-tolerant control for wind energy systems, offering insights into robust fault handling. For quarter-car T-S fuzzy suspension platforms, PSO is adopted to optimize fuzzy observer and LQR gains, while only abstract fault coefficients can be estimated [14]. For systems without full-state measurement, adaptive fuzzy output feedback FTC was designed, which requires full controller reconfiguration after actuator faults [15]. Finite-time disturbance observers are established to cope with actuator saturation and partial measurable signals, but faults are merged into lumped uncertainties without independent separation [16]. Zhou et al. [17] developed an adaptive fault-tolerant attitude tracking controller using a neural network disturbance observer, which handles actuator faults and input saturation simultaneously. Li et al. [18] designed an active fault-tolerant control scheme for four-wheel independent steering systems using a multi-agent approach.

Despite these advances, most existing FTC methods rely on abstract fault gain modeling or require reconfiguring the controller after faults occur, which increases computational complexity and limits their practical applicability. This motivates us to develop a more efficient and practical fault-tolerant control scheme for active suspension systems.

Presently, the vast majority of active suspension control research based on LQR operates under the idealized assumption that all system components, particularly the core actuators, remain in perfect working order [19,20]. However, during actual operation, actuators are highly susceptible to failures such as gain decay and output deviation due to prolonged exposure to impacts and wear from complex road conditions. Once an actuator fails, the performance of LQR controllers designed based on intact models will significantly deteriorate. Not only will they fail to achieve the intended control outcomes, but they may even aggravate system response, posing a threat to driving safety [21,22]. To enhance system reliability, research into fault-tolerant control has emerged. Active fault-tolerant control involves redesigning the controller based on fault diagnosis results after a fault occurs, thereby maintaining system stability while preserving performance levels close to those in normal conditions [23,24]. Among these approaches, active fault-tolerant control based on fault estimation, which dynamically adjusts control strategies, represents the current mainstream direction. Reference [25] employs an adaptive observer for online estimation of actuator gain fault factors; Reference [26] utilizes a sliding-mode observer to achieve fault detection and isolation. In [27], Sun et al. designed a highly reliable fault-tolerant controller accounting for actuator failures, parameter uncertainties, and external disturbances based on adaptive compensation control methods. Yang and Chen [28] proposed an active fault-tolerant control strategy based on sensor signal reconstruction to enhance the performance of active vehicle suspension control with sensor failures. Morato et al. [29] designed a fault-tolerant dynamic output feedback controller for semi-active suspension systems, validating its performance through simulation. In [30], Liu et al. proposed an active fault-tolerant controller based on an observer for active vehicle suspension systems where actuator faults manifest as non-zero offset faults. Yan Shuai et al. [31] proposed a combined fault-tolerant and fault diagnosis scheme to address failures in multiple actuators. These studies have laid a crucial foundation for fault-tolerant suspension control. However, most existing studies mainly estimate abstract fault gains or modes and cannot directly reflect the dynamic influence of faults on system states. In addition, few studies provide quantitative estimation of actual fault forces. To solve these problems, this paper establishes a quarter-car active suspension model with multiple practical actuator faults and proposes an adaptive fault observer to estimate system states and actual fault forces simultaneously. On this basis, a unified LQR-based fault-tolerant control scheme is constructed without reconfiguring the controller, which significantly improves system stability and ride comfort under actuator faults.

To address the aforementioned research gaps, this paper investigates a quarter-car active suspension system, considering a fault scenario where the actuator experiences force loss immediately upon initialization. The core contribution lies in designing an adaptive fault observer. This observer not only directly estimates the actual fault force of the actuator with high precision [32], but also continuously and accurately estimates the critical state variables of the system under both faulted and fault-tolerant conditions. Building upon this, the paper systematically compares and analyzes the dynamic performance of the system under normal, fault, and fault-tolerant states within a unified LQR control framework. Simulation results demonstrate that the proposed adaptive observer achieves precise estimation of both fault forces and state variables. Concurrently, the LQR controller provides effective control throughout the entire process. The fault-tolerant strategy, based on estimated information, significantly enhances performance during fault conditions, substantially restoring key suspension metrics. This validates the effectiveness of the proposed approach.

Compared with the existing fault-tolerant control methods for active suspension systems, the main novelties and contributions of this paper are summarized as follows:

(1) Direct and quantitative fault force estimation: Unlike most existing studies that only estimate abstract fault gains or modes, this paper designs an adaptive fault observer to directly and quantitatively estimate the actual fault force of the actuator, which can more intuitively reflect the dynamic influence of faults on system states.

(2) Unified LQR-based fault-tolerant control framework without controller reconfiguration: Different from traditional fault-tolerant control schemes that need to redesign the controller after fault occurrence, this paper constructs a unified fault-tolerant control framework based on LQR control, which maintains the same controller structure and only introduces fault compensation, thus reducing computational complexity and improving real-time performance.

(3) Simultaneous state and fault estimation: The proposed observer can achieve high-precision online estimation of both system critical state variables and actuator fault forces at the same time, which is more practical for actual vehicle suspension applications.

The structure of this paper is arranged as follows: Section 1 introduces the research background and scope; Section 2 establishes a quarter-car active suspension model and an actuator fault model; Section 3 details the design process of the LQR controller and adaptive fault observer; Section 4 verifies the estimation accuracy of the observer through simulation and compares the suspension performance under three conditions; Section 5 summarizes the entire paper.

## 2. Suspension System Dynamics Model

**Figure 1:**
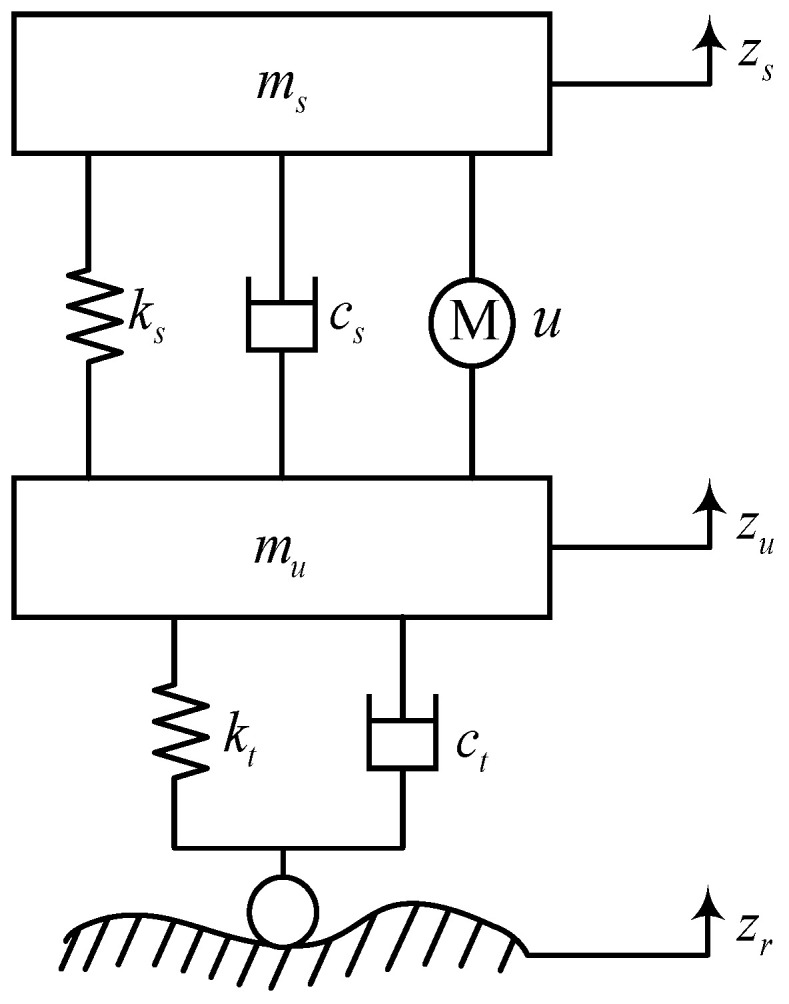
Structure diagram of the quarter-car suspension model.

The quarter-car active suspension system has been extensively studied due to its simple structure and representative dynamic characteristics. Its mechanical structure is shown in Figure 1, where $m_{s}$ represents the sprung mass, $m_{u}$ denotes the unsprung mass, $k_{s}$ and $k_{t}$ indicate the stiffness coefficients of the suspension and tire respectively, $c_{s}$ and $c_{t}$ denote the damping coefficients of the suspension and tire respectively, the force provided by the actuator motor is defined as u, and $z_{s}$, $z_{u}$, and $z_{r}$ represent the displacements of the sprung mass, unsprung mass, and uneven road surface respectively. The dynamic equations for this two-degrees-of-freedom suspension are given by [33]

(1) $$\left\{\begin{array}{c} u-k_{s}(z_{s}-z_{u})-c_{s}({\dot{z}}_{s}-{\dot{z}}_{u})=m_{s}\ddot{z}\\ k_{s}(z_{s}-z_{u})+c_{s}({\dot{z}}_{s}-{\dot{z}}_{u})-k_{t}(z_{u}-z_{r})-c_{t}({\dot{z}}_{u}-{\dot{z}}_{r})-u=m_{u}{\ddot{z}}_{u} \end{array}\right.$$

To obtain the state-space form of the system, the following state variables are defined:

(2) $$X_{1}=x_{1}\left(t\right)={\dot{z}}_{s}\left(t\right)$$

(3) $$X_{2}=x_{2}\left(t\right)={\dot{z}}_{u}\left(t\right)$$

(4) $$X_{3}=x_{3}\left(t\right)=z_{s}\left(t\right)-z_{u}\left(t\right)$$

(5) $$X_{4}=x_{4}\left(t\right)=z_{u}\left(t\right)-z_{r}\left(t\right)$$

Select road surface speed as the disturbance input, that is, $W\left(t\right)={\dot{z}}_{r}\left(t\right)$.

Define the state vector:

(6) $$\begin{array}{l} X\left(t\right)={\left[\begin{array}{cccc} X_{1} & X_{2} & X_{3} & X_{4} \end{array}\right]}^{T}\\ ={\left[\begin{array}{cccc} x_{1}\left(t\right) & x_{2}\left(t\right) & x_{3}\left(t\right) & x_{4}\left(t\right) \end{array}\right]}^{T} \end{array}$$

The state-space representation of the active suspension system can then be derived:

(7) $$\dot{X}\left(t\right)=AX\left(t\right)+BU\left(t\right)+GW\left(t\right)$$

The specific forms of the coefficient matrices A, B, G, and U are as follows:

$$\begin{array}{c} A=\left[\begin{array}{cccc} -\frac{c_{s}}{m_{s}} & \frac{c_{s}}{m_{s}} & -\frac{k_{s}}{m_{s}} & 0\\ \frac{c_{s}}{m_{u}} & -\frac{c_{s}+c_{t}}{m_{u}} & \frac{k_{s}}{m_{u}} & -\frac{k_{t}}{m_{u}}\\ 1 & -1 & 0 & 0\\ 0 & 1 & 0 & 0 \end{array}\right],\;B=\left[\begin{array}{c} \frac{1}{m_{s}}\\ -\frac{1}{m_{u}}\\ 0\\ 0 \end{array}\right],\;G=\left[\begin{array}{c} 0\\ \frac{c_{t}}{m_{u}}\\ 0\\ -1 \end{array}\right],\;\\ U=\left[u\right]. \end{array}$$

In accordance with the performance requirements of the suspension system, the control outputs are selected as follows:

(8) $$Y\left(t\right)={\left[\begin{array}{ccc} {\ddot{z}}_{s}\left(t\right) & z_{s}\left(t\right)-z_{u}\left(t\right) & z_{u}\left(t\right)-z_{r}\left(t\right) \end{array}\right]}^{T}$$

For convenience of measurement, let the measurement output be denoted as $Z\left(t\right)={\left[\begin{array}{cccc} X_{1} & X_{2} & X_{3} & X_{4} \end{array}\right]}^{T}$.

Therefore, the vehicle suspension system can be described by the following formula:

(9) $$\left\{\begin{array}{c} \dot{X}\left(t\right)=AX\left(t\right)+BU\left(t\right)+GW\left(t\right)\\ Y\left(t\right)=CX\left(t\right)+DU\left(t\right)\\ Z\left(t\right)=C_{z}X\left(t\right) \end{array}\right.$$

where

$$C=\left[\begin{array}{cccc} -\frac{c_{s}}{m_{s}} & \frac{c_{s}}{m_{s}} & -\frac{k_{s}}{m_{s}} & 0\\ 0 & 0 & 1 & 0\\ 0 & 0 & 0 & 1 \end{array}\right],\;D={\left[\begin{array}{ccc} \frac{1}{m_{s}} & 0 & 0 \end{array}\right]}^{T},\;C_{z}=\left[\begin{array}{cccc} 1 & 0 & 0 & 0\\ 0 & 1 & 0 & 0\\ 0 & 0 & 1 & 0\\ 0 & 0 & 0 & 1 \end{array}\right].$$

Due to factors such as the increased mileage or the abnormal usage of the vehicle, the actuator of the active suspension system may exhibit the following faults: gain variation, steady-state error, and seizure. In order to consider these actuator faults, the following operations are performed:

Assume the aforementioned faults occur in the jth actuator, which can be expressed as

(10) $$u_{jf}=\delta_{j}u_{j}+\beta_{j}$$

where $u_{jf}$ denotes the output of the jth actuator with fault; $u_{j}$ denotes the output of the jth actuator without fault; $\delta_{j}$ denotes the error severity of the jth actuator; and $\beta_{j}\in \left(0,u_{\max}\right]$ denotes the jth actuator being jammed or exhibiting a constant deviation. Different values of $\delta_{j}$ and $\beta_{j}$ represent different fault modes, which are detailed in Table 1.

According to (10), the output vector $U_{f}$ of the fault actuator can be described as

(11) $$\begin{array}{cc} U_{f} & =\left[u_{f}\right]=\left[\delta u+\beta \right]\\ & =\left[u\right]+\left[\left(\delta -1\right)u+\beta \right]\\ & =U+F \end{array}$$

where F denotes the actuator fault value, which can be expressed as

(12) $$F=\left[\left(\delta -1\right)u+\beta \right]$$

Note: This quarter-car model contains only one single actuator, so the subscript j in Equation (10) refers to this unique actuator. Here $U=\left[u_{j}\right]$; the uppercase vector notation is only adopted to keep consistent with the vector-based state-space expressions in Equations (9) and (13), rather than describing multiple actuators.

Thus, the quarter-car model of the active suspension system with actuator failure is obtained:

(13) $$\left\{\begin{array}{c} \dot{X}=AX+B\left(U+F\right)+GW\\ Y=CX+D\left(U+F\right)\\ Z=C_{z}X \end{array}\right.$$

## 3. Fault-Tolerant Controller Design for Suspension Systems Based on Fault Estimation and Compensation

### 3.1. The System Architecture of the Fault-Tolerant Control Scheme

**Figure 2:**
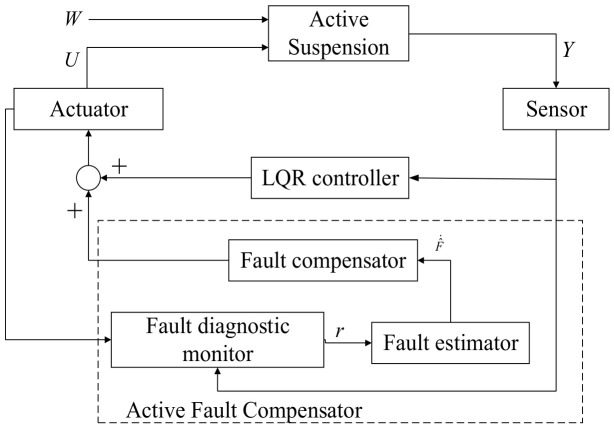
The system architecture of the fault-tolerant control for active suspension systems.

The block diagram of the fault-tolerant control for suspension systems based on the fault estimation and compensation is shown in Figure 2. It can be seen that the fault-tolerant control system is mainly composed of two components: an LQR controller and an Actuator Fault Compensator (AFC), which comprises a fault diagnosis observer, a fault estimator, and a fault compensator.

When the actuator works normally, the LQR controller ensures satisfactory control performance. On the other hand, when the actuator has some faults, the fault diagnosis observer calculates a fault residual error r according to the actuator state and system output and then sends the obtained fault residual error to the fault estimator. Then, the fault amplitude is calculated by the fault estimator based on the fault residual error and the obtained fault amplitude is sent to the fault compensator. Then, the fault compensator calculates the compensating value, and an Actuator Fault Compensator is obtained. In the end, the active fault-tolerant control is realized by combining the output signals of the AFC and LQR controller.

### 3.2. LQR Controller Design for the Active Suspension System

The purpose is to design an effective LQR controller such that the impact of the disturbance input W on the control output Y is minimized, thereby effectively suppressing the influences from the road surface irregularity disturbances on the upspring mass.

It is well known that LQR control is an optimal control strategy [34]. In this work, a quadratic performance index for active suspension systems is employed, and the vehicle ride comfort, handling stability, and energy consumption characteristics are comprehensively optimized. In order to improve the control performance, the vertical body acceleration ${\ddot{z}}_{s}$, suspension working space $\left(z_{s}-z_{u}\right)$, dynamic tire deflection $\left(z_{u}-z_{r}\right)$, and control input u are selected as system evaluation indicators. These indicators are chosen to comprehensively balance ride comfort, suspension stroke limitation, tire road-holding ability, and control energy consumption, which are the most critical performance requirements for active suspension systems. Then, a state regulator can be obtained based on optimal control theory, and its performance index is expressed as

(14) $$J=\underset{T\to \infty }{\lim}\frac{1}{T}\int_{0}^{T}\left[q_{1}{\ddot{z}}_{s}^{2}+q_{2}{\left(z_{s}-z_{u}\right)}^{2}+q_{3}{\left(z_{u}-z_{r}\right)}^{2}+q_{4}\cdot u^{2}\right]dt$$

where $q_{1}$, $q_{2}$, $q_{3}$, and $q_{4}$ denote the weighting coefficients of the performance evaluation metrics.

The four scalar weighting coefficients $q_{1}$, $q_{2}$, $q_{3}$, and $q_{4}$ in Equation (14) correspond to different suspension dynamic performance constraints. To simplify multi-objective performance adjustment, we assemble these coefficients into a three-dimensional diagonal output weighting matrix $Q_{1}$, which matches the three output penalty terms contained in the cost function:

$$Q_{1}=diag(q_{1},q_{2},q_{3})=\left[\begin{array}{ccc} 0.6 & 0 & 0\\ 0 & 0.01 & 0\\ 0 & 0 & 12500 \end{array}\right],\;q_{4}=1\times 10^{-6},$$

where the LQR state-related matrices satisfy $Q=C^{T}Q_{1}C$, $R=D^{T}Q_{1}D+q_{4}$, $N=C^{T}Q_{1}D$.

Note: $Q_{1}$ is the 3 × 3 weighting matrix corresponding to three output penalty terms in Equation (14). The state weighting matrix Q, input weighting matrix R and cross-term matrix N used in Equation (15) are not directly preset, and are calculated from $Q_{1}$ via the above transformation relations derived from the cost function. Such mapping converts the three-dimensional $Q_{1}$ into the four-dimensional state weighting matrix Q matching the dimension of four-dimensional state vector X, where $Q\in R^{4\times 4}$, $R\in R^{1\times 1}$, $N\in R^{4\times 1}$.

After transforming the low-dimensional output weighting matrix $Q_{1}$ to match the dimension of the four-dimensional state vector via the above mapping relations, we obtain the standard infinite-horizon quadratic LQR cost function, as expressed in Equation (15).

(15) $$J=\underset{T\to \infty }{\lim}\frac{1}{T}\int_{0}^{T}\left(X^{T}QX+U^{T}RU+2X^{T}NU\right)dt$$

where Q, R, and N are the weighting matrices of the system state variables, control input variables, and their interaction terms, respectively.

The weighting matrix Q is used to penalize system state deviations, and the weighting matrix R is used to limit the control energy and avoid actuator saturation. The trade-off between system performance and control effort is realized by adjusting Q and R.

Specifically, a larger $q_{1}$ suppresses vertical body acceleration to improve ride comfort; increasing $q_{2}$ limits suspension working stroke to avoid mechanical collision between the sprung and unsprung masses; a higher $q_{3}$ strengthens tire dynamic deflection constraint and guarantees tire road-holding performance; and the coefficient $q_{4}$ restricts the amplitude of control input to prevent actuator saturation. In practical parameter tuning, we prioritize ride comfort while satisfying hard constraints on suspension displacement and tire dynamic load, which reflects the multi-objective trade-off characteristics of active suspension control.

By employing the LQR solver within the MATLAB Control System Toolbox to solve the algebraic Riccati equation $\left[K,S,E\right]=lqr\left(A,B,Q,R,N\right)$, the feedback gain K can be obtained as the sought-after optimal feedback control gain. Furthermore, by altering the values of $q_{1}$, $q_{2}$, $q_{3}$, and $q_{4}$ while iteratively refining the design, an LQR controller with satisfactory control performance can be achieved. The elements in Q and R are determined through extensive simulation experiments and engineering experience, prioritizing ride comfort while satisfying suspension displacement constraints and tire dynamic load limitations. Considering the time-varying non-stationary property of practical road excitation and the core research focusing on actuator fault estimation and active compensation rather than stochastic optimal control, LQR instead of LQG is selected as the baseline controller in this paper.

### 3.3. Fault Estimation of the Active Suspension System Actuator

In practice, certain system faults are difficult to measure directly using physical sensors. Therefore, designing fault estimators is very important for the system to obtain satisfactory performance [35]. Here, based on past experience, the following adaptive fault estimator is established to detect the fault occurring in the active suspension system actuator:

(16) $$\left\{\begin{array}{c} \dot{\hat{X}}=A\hat{X}+B\left(U+\hat{F}\right)-L\left(\hat{Z}-Z\right)\\ \hat{Z}=C_{Z}\hat{X} \end{array}\right.$$

where $\hat{X}$ denotes the estimate of the state X; $\hat{Z}$ denotes the estimate of the measurement output Z; $\hat{F}$ denotes the estimate of the actuator fault vector F; and L denotes the observer gain matrix regulating state estimation error dynamics.

The adaptive learning law in Equation (17) suppresses steady-state bias and guarantees convergent fault estimation.

All estimation errors are proven convergent via LMI constraints in Theorem 1 of Section 3.5.

The estimate errors are defined as $e_{x}=\hat{X}-X$, $e_{F}=\hat{F}-F$, and $r=\hat{Z}-Z$, where $H\in R^{1\times 3}$, $\dot{\hat{F}}\in R^{1\times 1}$, and $r\in R^{3\times 1}$, and a fault estimation algorithm is given as follows:

(17) $$\dot{\hat{F}}=-Hr$$

where H is the learning gain matrix adjusting fault estimation convergence speed. Then, the dynamic error equation may be expressed as

(18) $$\left\{\begin{array}{c} {\dot{e}}_{x}=\dot{\hat{X}}-\dot{X}=\left(A-LC_{z}\right)e_{x}-GW+Be_{F}\\ {\dot{e}}_{F}=\dot{\hat{F}}-\dot{F}=-HC_{z}e_{x}-\dot{F} \end{array}\right.$$

Then, the following fault estimation error system formed by (18) is obtained:

(19) $$\dot{\bar{e}}=\left(\bar{A}-\bar{L}\bar{C}\right)\bar{e}-\bar{B}w$$

where

$$\bar{e}=\left[\begin{array}{c} e_{x}\\ e_{F} \end{array}\right];\;w=\left[\begin{array}{c} W\\ \dot{F} \end{array}\right];\;\bar{A}=\left[\begin{array}{cc} A & B\\ 0 & 0 \end{array}\right];\;\bar{L}=\left[\begin{array}{c} L\\ H \end{array}\right];\;\bar{C}=\left[\begin{array}{cc} C_{z} & 0 \end{array}\right];\;\bar{B}=\left[\begin{array}{cc} G & 0\\ 0 & I \end{array}\right].$$

Here I denotes the identity matrix, and $A\in R^{4\times 4}$, $B\in R^{4\times 1}$, $G\in R^{4\times 1}$, $C_{Z}\in R^{3\times 4}$, $L\in R^{4\times 3}$, $\bar{A}\in R^{5\times 5}$, $\bar{B}\in R^{5\times 2}$, $\bar{C}\in R^{3\times 5}$, and $\bar{L}\in R^{5\times 3}$.

The fault observer is designed with two core objectives: ensuring the stability of the augmented error system and achieving asymptotic convergence of state and fault estimation errors. To improve the estimate accuracy of the fault estimator, it is essential to design appropriate observer gain matrix L and fault learning rate H such that the augmented error system in (19) satisfies the following conditions: (1) the system is stable and meets the LQR performance criterion ${\left\|\bar{e}\right\|}_{2}\leq \gamma {\left\|w\right\|}_{2}$; (2) the system state vector gradually converges towards zero.

Let the Lyapunov function be denoted by $V={\bar{e}}^{T}P\bar{e}$, then

(20) $$\begin{array}{l} \dot{V}={\dot{\bar{e}}}^{T}P\bar{e}+{\bar{e}}^{T}P\dot{\bar{e}}=\\ {\bar{e}}^{T}\left(P\left(\bar{A}-\bar{L}\bar{C}\right)+{\left(\bar{A}-\bar{L}\bar{C}\right)}^{T}P\right)\bar{e}-2{\bar{e}}^{T}P\bar{B}w \end{array}$$

Treating w as the disturbance input, define the LQR performance metric as J, and the fault estimator is satisfied [36].

(21) $$\frac{\sqrt{\int_{0}^{t}{\bar{e}}^{T}\bar{e}dt}}{\sqrt{\int_{0}^{t}w^{T}wdt}}\leq \gamma$$

Equation (21) is equivalent to

(22) $$\sqrt{\int_{0}^{t}\left(\gamma^{-2}{\bar{e}}^{T}\bar{e}-w^{T}w\right)dt}\leq 0$$

By considering (20), (22) and $\dot{V}<0$, it yields

(23) $$\left[\begin{array}{cc} P\left(\bar{A}-\bar{L}\bar{C}\right)+{\left(\bar{A}-\bar{L}\bar{C}\right)}^{T}P+\gamma^{-2}I & -P\bar{B}\\ {}* & -I \end{array}\right]<0$$

If (23) is solvable, the augmented error system is stable and the LQR performance is satisfied, obviously. Equation (23) ensures that the system satisfies Condition 1. In order to ensure the system satisfies Condition 2, the system state vector $\bar{e}$ gradually converges towards zero, and the following regional pole placement lemma is introduced.

**Lemma** **1** ([37])**.** *The eigenvalue* z *of the state matrix* $A\in R^{n\times n}$ *of a given system lies in the region* $D_{\alpha }\left(z\in C,Re\left(z\right)\leq -\alpha \right)\left(\alpha >0\right)$ *if and only if there exists a symmetric positive definite matrix* $P\in R^{n\times n}$ *such that*

(24) $$PA+A^{T}P+2\alpha P<0$$

Region $D_{\alpha }$ is a half-plane region that ensures the state responses exhibit an attenuation rate α.

By Lemma 1, substituting the state matrix $\bar{A}-\bar{L}\bar{C}$ of the augmented error system for the matrix A in (24) yields

(25) $$P\left(\bar{A}-\bar{L}\bar{C}\right)+{\left(\bar{A}-\bar{L}\bar{C}\right)}^{T}P+2\alpha P<0$$

thereby ensuring that the state vector $\bar{e}$ of the extended error system converges with a stable margin α and asymptotically approaches zero.

By combining (23) and (25), and taking $N=P\bar{L}$, it is obtained that

(26) $$\left\{\begin{array}{c} \left[\begin{array}{cc} P\bar{A}+{\bar{A}}^{T}P-N\bar{C}-{\bar{C}}^{T}N^{T}+\gamma^{-2}I & -P\bar{B}\\ {}* & -I \end{array}\right]<0\\ \begin{array}{l} P\bar{A}+{\bar{A}}^{T}P-N\bar{C}-{\bar{C}}^{T}N^{T}+2\alpha P<0\\ P>0 \end{array} \end{array}\right.$$

By solving the matrix inequality system (26), a set of feasible solutions $P^{*}$ and $N^{*}$ satisfying the design objectives can be obtained. Furthermore, the feasible solution ${\bar{L}}^{*}$ for the matrix $\bar{L}$, composed of the observer gain matrix L and the fault learning rate H, is given by

(27) $${\bar{L}}^{*}={\left(P^{*}\right)}^{-1}N^{*}$$

The observer integrates multi-source measurements including sprung mass acceleration and suspension deflection. Multi-sensor fusion effectively suppresses false alarms triggered by a single poorly calibrated sensor and distinguishes real actuator faults from sensor drift errors.

### 3.4. Actuator Fault Compensation Design

To ensure the active suspension system maintains stability and acceptable performance during fault conditions, a fault compensator is designed based on online estimation of actuator faults, subject to the following assumption. Suppose there exists a matrix $B^{*}$ of appropriate dimension such that the following equation holds:

(28) $$\left(I_{n}-BB^{*}\right)B=0$$

Upon calculation, the expression for $B^{*}$ satisfying (28) is

(29) $$B^{*}={\left(B^{T}B\right)}^{-1}B^{T}$$

Then, a compensator based on actuator fault estimation information is designed as follows:

(30) $$U_{afc}=-B^{*}B\dot{\hat{F}}$$

The fault-tolerant controller, comprising an LQR controller and compensator in fault mode, is defined as follows:

(31) $$\left\{\begin{array}{c} {\dot{X}}_{k}=A_{k}X_{k}+B_{k}Z\\ U=C_{k}X_{k}+D_{k}Z-B^{*}B\dot{\hat{F}} \end{array}\right.$$

Applying (31) to the suspension system (13), the closed-loop system with the fault-tolerant controller can be described as

(32) $$\dot{X}=\left(A+BD_{k}C_{y}\right)X+GW+BC_{k}X_{k}+Be_{F}$$

Applying Equation (32) to the active suspension system (9), the closed-loop system with the normal LQR output feedback controller can be expressed as

(33) $$\dot{X}=\left(A+BD_{k}C_{y}\right)X+GW+BC_{k}X_{k}$$

where $D_{k}\in R^{1\times 3}$, $C_{y}\in R^{3\times 4}$, $G\in R^{4\times 2}$, $W\in R^{2\times 1}$, $C_{k}\in R^{3\times 4}$, $X\in R^{4\times 1}$, and $X_{k}\in R^{4\times 1}$. The rigorous stability proof of the closed-loop fault-tolerant system is presented in Section 3.5.

### 3.5. Stability Analysis

In this subsection, the stability of the augmented error system and the closed-loop fault-tolerant control system is rigorously proved.

**Assumption** **1.** *The external road disturbance* w *is bounded,* i.e.*,* $\left\|w\right\|\leq w_{\max}$*, where* $w_{\max}$ *is a positive constant.*

**Theorem** **1.** *Consider the faulty suspension system given in Equation (13), the adaptive fault observer designed in Equation (16), and the fault-tolerant controller presented in Equation (31). If there exists a symmetric positive definite matrix* $P=P^{T}>0$ *such that* ${\left(\bar{A}\right)}^{T}P+P\bar{A}<0$*, where matrices* $\bar{A}$ *and vectors* $\bar{e}$ *follow the definitions presented in Equation (19), the feasible solution of matrix* P *can be calculated via the LMI toolbox as given in Equation (26). Then the augmented fault estimation error dynamic described in Equation (19) is globally asymptotically stable. Accordingly, the state estimation error* e *converges to zero asymptotically, the closed-loop fault-tolerant dynamic system expressed in Equation (32) is asymptotically stable, and all physical and estimated signals within the closed-loop control structure satisfy the uniform ultimate boundedness property against bounded external disturbance* w*.*

**Proof.**  From the previous derivation, the dynamics of augmented error has been given in Equation (19). Construct the quadratic Lyapunov functional:

Construct the quadratic Lyapunov candidate function:

(34) $$\left\{\begin{array}{c} V\left(\bar{e}\right)={\bar{e}}^{T}P\bar{e}\\ P=P^{T}>0 \end{array}\right.$$

Differentiating V along the solution of Equation (19) yields

(35) $$\begin{array}{cc} \dot{V} & ={\dot{\bar{e}}}^{T}P\bar{e}+{\bar{e}}^{T}P\dot{\bar{e}}\\ & ={\bar{e}}^{T}\left({\bar{A}}^{T}P+P\bar{A}\right)\bar{e}+2{\bar{e}}^{T}P\bar{D}w \end{array}$$

Based on the LMI condition ${\bar{A}}^{T}P+P\bar{A}<0$, the quadratic term ${\bar{e}}^{T}\left({\bar{A}}^{T}P+P\bar{A}\right)\bar{e}$ is negative definite, which gives

(36) $$\dot{V}\leq 2\left\|P\bar{D}\right\|\cdot \left\|\bar{e}\right\|\cdot \left\|w\right\|$$

In accordance with Assumption 1, the disturbance w is bounded. We can always find a positive constant ε such that $\dot{V}<0$ whenever $\left\|\bar{e}\right\|>\varepsilon$. Accordingly, the variable $\bar{e}$ tends to zero as time goes to infinity, which further yields the convergence of $e_{F}$ to zero.

Next, we investigate the closed-loop system described by Equation (32). The gain matrix is optimized via LQR such that $\left(A+BD_{k}C_{y}\right)$ is Hurwitz stable. Combined with the convergence of $e_{F}$ and boundedness of w, the closed-loop system is asymptotically stable.

Moreover, all involved signals X, $\hat{X}$, $X_{k}$, U, and $e_{F}$ are uniformly ultimately bounded. This completes the proof. □

Based on the real-time fault information from the proposed observer, an extra feedforward compensation signal is constructed to restructure the control law and offset the actuator efficiency loss. Such control reconfiguration guarantees the closed-loop stability of active suspension under actuator faults, which reflects the core reconfiguration property of the proposed fault-tolerant controller.

## 4. Simulation Results

In practical implementation, the road excitation can be indirectly obtained via tire displacement or wheel acceleration sensors installed on vehicle wheels to realize real-time data acquisition.

To validate the effectiveness of the proposed quarter-car active suspension fault-tolerant control strategy, each module depicted in the fault-tolerant control strategy block diagram (Figure 2) was implemented in MATLAB R2023a/Simulink. Following program development, simulations and analyses were conducted for active suspension fault estimation and fault-tolerant control. Parameters for the vehicle active suspension model and road surface model were selected based on the relevant literature.

### 4.1. Random Road Model

This paper employs random road surface excitation, whose power spectral density is described by the following power function form [38]:

(37) $$G_{xr}\left(n\right)=G_{xr}\left(n_{0}\right){\left(\frac{n}{n_{0}}\right)}^{-2}$$

In Equation (37), n represents the spatial frequency; $n_{0}$ denotes the reference spatial frequency, where $n_{0}=0.1\;m^{-1}$; and $G_{xr}\left(n_{0}\right)$ is the pavement unevenness coefficient.

Employing the filtered white noise method, the mathematical expression for establishing a time-domain random pavement model is as follows:

(38) $${\dot{Z}}_{r}\left(t\right)=-2\pi f_{\min}z_{r}\left(t\right)+2\pi n_{0}w\left(t\right)\sqrt{\left(G_{xr}\left(n_{0}\right)v\right)}$$

The lower time-domain cutoff frequency is $f_{\min}=v\cdot n_{\min}$; the lower spatial frequency cutoff is $n_{\min}=0.011\;m^{-1}$; $w\left(t\right)$ is zero-mean Gaussian white noise with constant power spectral density; and v is the vehicle speed.

**Figure 3:**
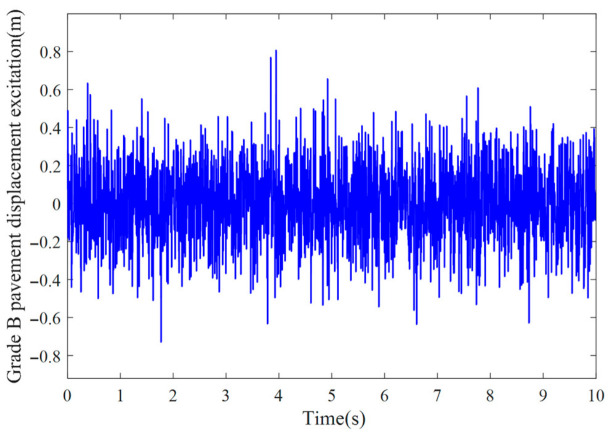
Time−domain response curve of Grade B pavement unevenness.

This paper uses grade B pavement as the reference condition, where the pavement unevenness coefficient $G_{xr}\left(n_{0}\right)=64\times 10^{-6}\;m^{3}$ and vehicle speed v=60 km/h are specified. Figure 3 shows the vertical displacement response curve of the wheel under random pavement excitation.

Next, the active suspension of a quarter-car is simulated. The suspension parameters are listed in Table 2 [39].

### 4.2. Fault Estimation Simulation Analysis

The design of the active suspension actuator fault value accounts for gain faults in the actuator, considering only an initial loss of 0.8 times the control force.

**Figure 4:**
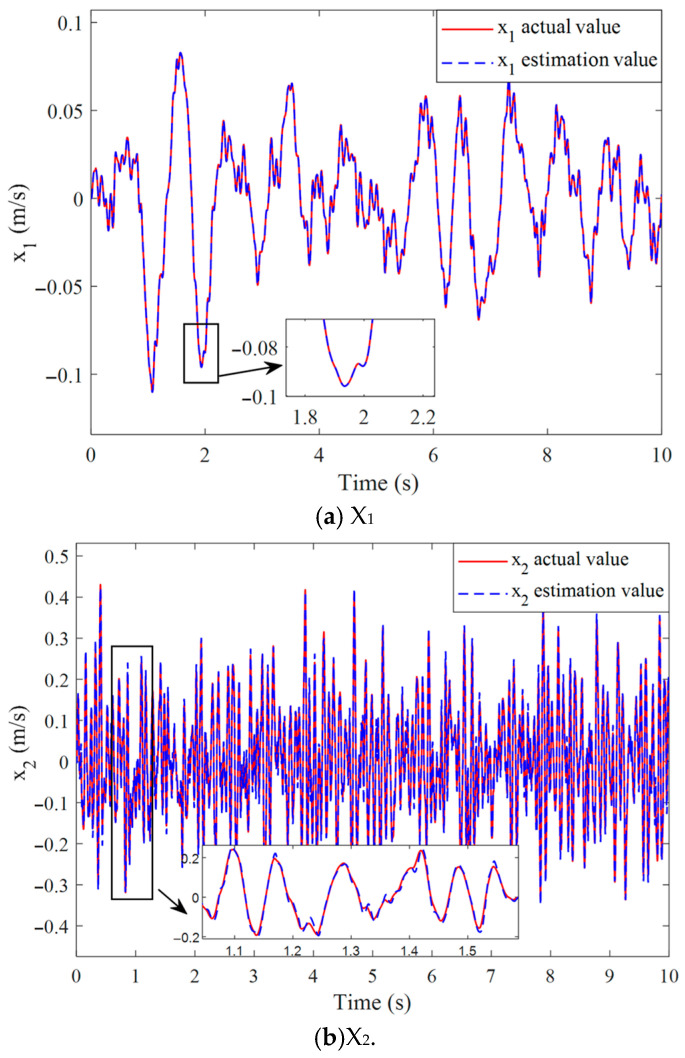

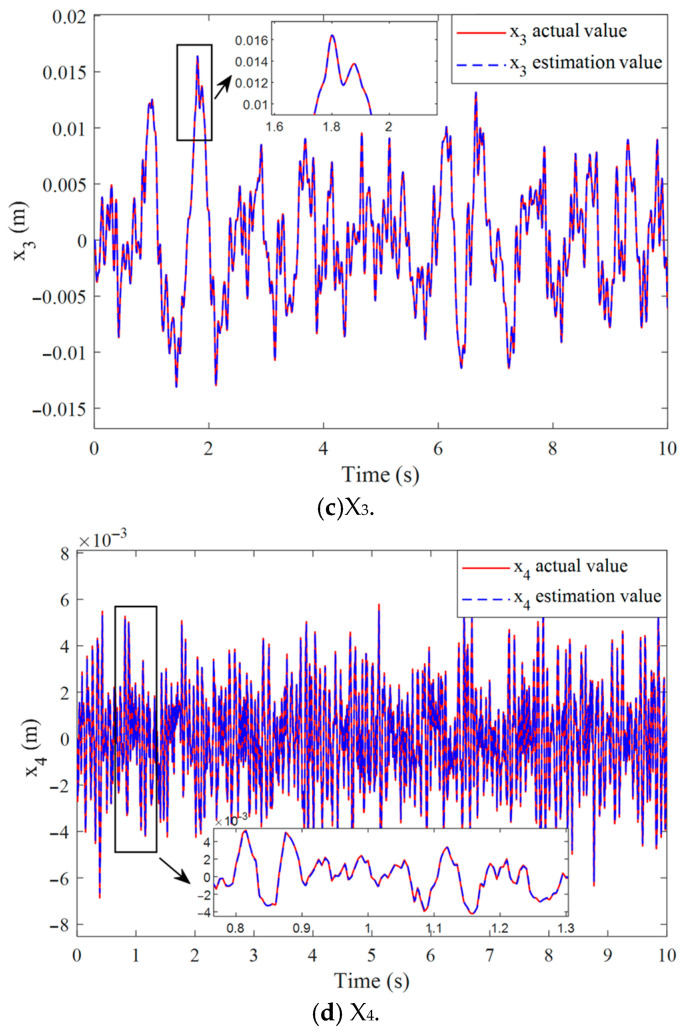
Estimation of state variables under fault conditions.

Estimation of state variables is performed as follows: First, each state variable X under fault conditions is estimated. The resulting estimation diagram is shown in Figure 4.

In this severe case with 80% actuator control force loss, the designed feedforward compensation restrains the excessive increase in sprung mass displacement and dynamic tire deflection effectively. Contrasted with the suspension without fault compensation, both ride comfort and road-holding performance are well preserved under near-total actuator failure.

**Figure 5:**
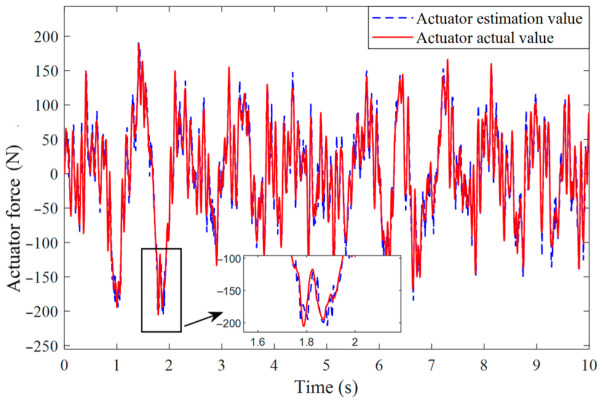
Actuator force estimation under fault conditions.

As shown in Figure 4, under fault conditions, the proposed adaptive fault observer exhibits fast convergence and achieves high-precision estimation of the system state variables. The estimation error between the actual and estimated values is sufficiently small, which verifies the effectiveness of the observer. The estimation of the fault force is illustrated in Figure 5.

**Figure 6:**
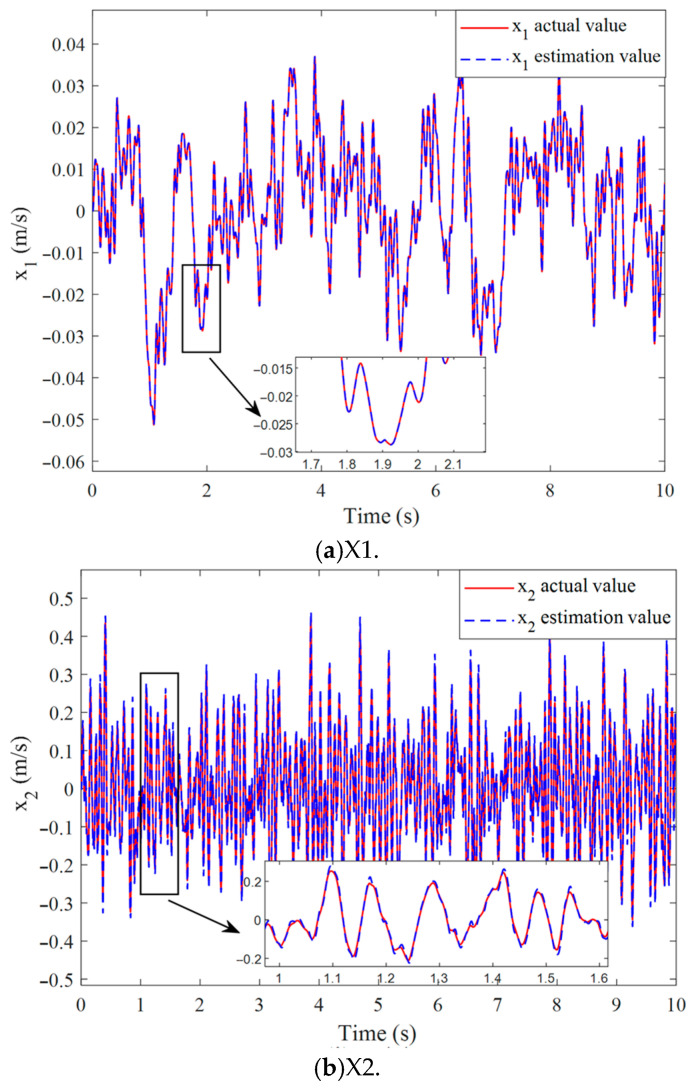

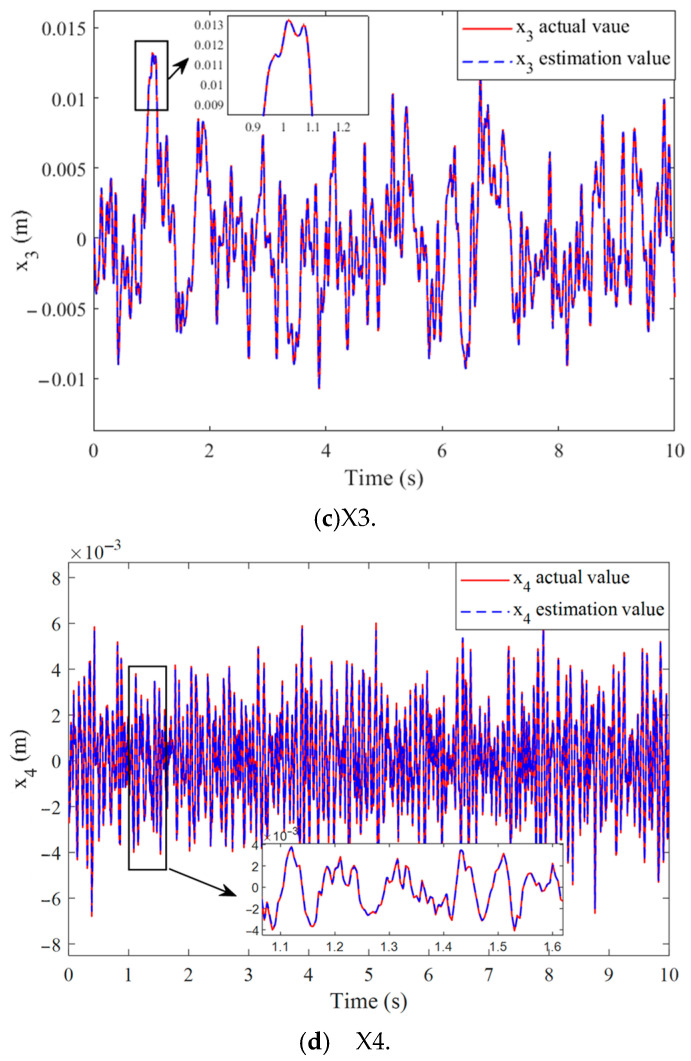
Estimation of state variables under fault-tolerant conditions.

As shown in Figure 5, highly accurate fault estimation is achieved under fault conditions. Subsequently, the state variables under fault-tolerant conditions were estimated, with the results illustrated in Figure 6.

**Figure 7:**
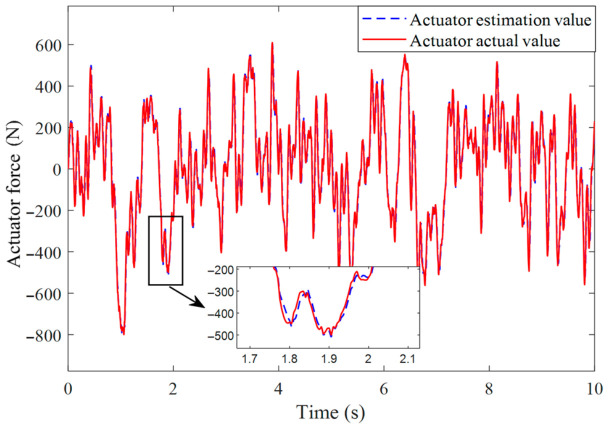
Actuator force estimation under fault-tolerant conditions.

As shown in Figure 6, it can be seen that our estimation of the state variable is highly accurate. The observer can still track the actual state variables stably and accurately under fault-tolerant working conditions. Next, we estimate the actual output force of the actuator under fault-tolerant conditions, with the estimation results shown in Figure 7.

Figure 5 presents the comparison between actual and estimated actuator output under the fault condition, while Figure 7 shows the corresponding comparison under the fault-tolerant condition. These two figures are used to verify the estimation accuracy of the developed adaptive observer.

**Figure 8:**
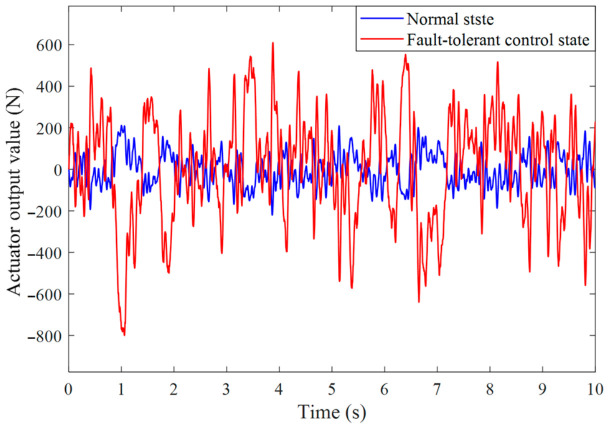
Actuator output forces under normal and fault-tolerant conditions.

Figure 8 is supplemented to compare actuator output force under the normal state and fault-tolerant compensated state. The output range from −800 N to 600 N is naturally formed under the simulation condition of grade B random road excitation plus 80% actuator control force loss, instead of the inherent rated saturation limit of the physical actuator hardware.

### 4.3. Simulation of Active Suspension Control Systems

**Figure 9:**
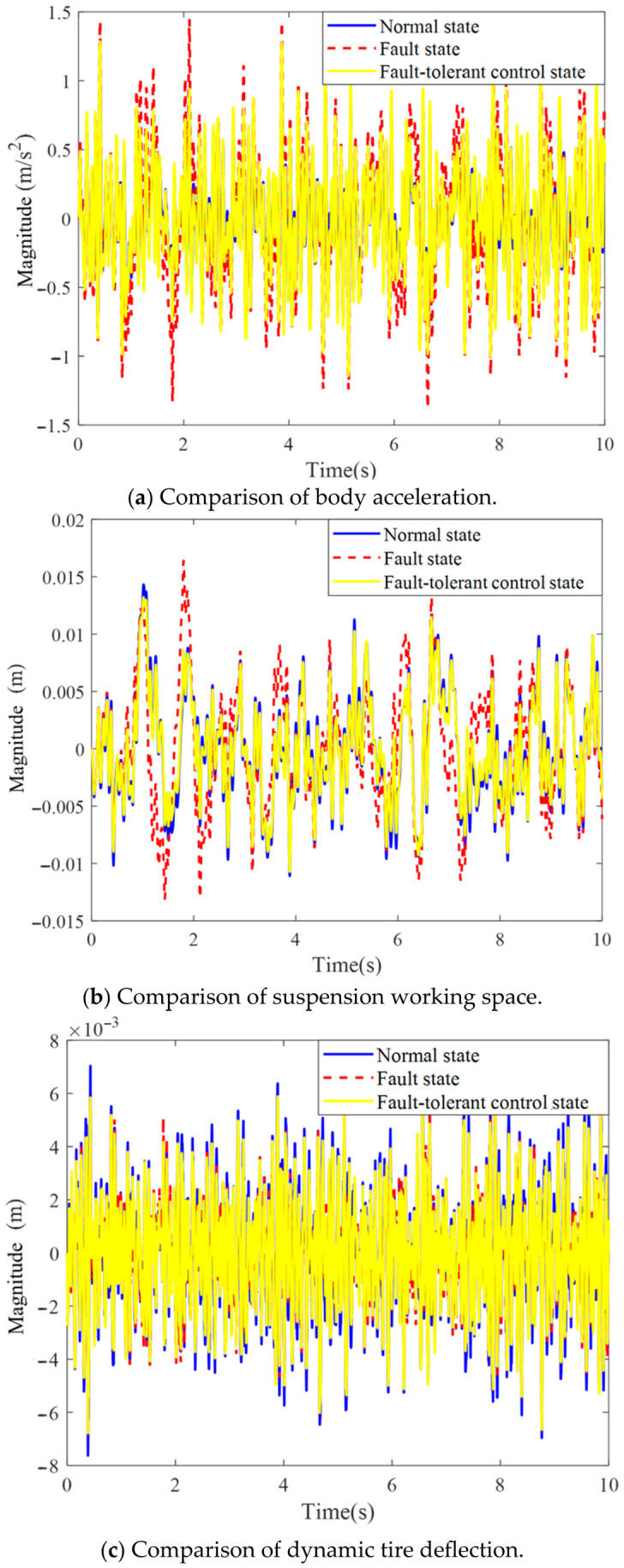
Comparison of suspension performance.

To validate the effectiveness of the designed fault-tolerant control, active fault-tolerant control for the suspension actuator was achieved through the combined action of the AFC and LQR weighted output feedback controllers. This was based on the accurate estimation of the actuator fault magnitude by the fault estimator using an adaptive observer. The suspension actuator was set to experience a 0.8-fold gain fault at the initial moment. The simulation was conducted at a vehicle speed of 60 km/h on a road of grade B, with a total simulation time of 10 s. The simulation results for the performance metrics of the suspension actuator under the three conditions are shown in Figure 8 on a B-grade road surface for a total simulation duration of 10 s. The simulation results for the suspension’s performance indexes under normal state (N-S), fault state (F-S), and fault-tolerant control state (F-T-C-S) are shown in Figure 9. The fault tolerance effect (F-T-E), as demonstrated by comparing the fault-tolerant control state with the fault state, is detailed in Table 3.

As shown in Table 3, within the 0~10 s interval, the fault tolerance effect for body vertical acceleration is 14.59%, the suspension working space fault tolerance effect is 16.98%, and the dynamic tire deflection fault tolerance effect shows a slight deterioration of 5%. However, the fault-tolerant control still yields better results than the normal state. Specifically, the fault-tolerant control effectively suppresses the increase in body acceleration and suspension working space caused by the actuator fault, ensuring both ride comfort and structural safety. Simulation results indicate that under the designed fault-tolerant control strategy, the fault-tolerant performance of the quarter-car active suspension is effectively enhanced. This simultaneously improves vehicle ride comfort and handling stability while ensuring safety, demonstrating the effectiveness of the fault-tolerant control strategy.

**Figure 10:**
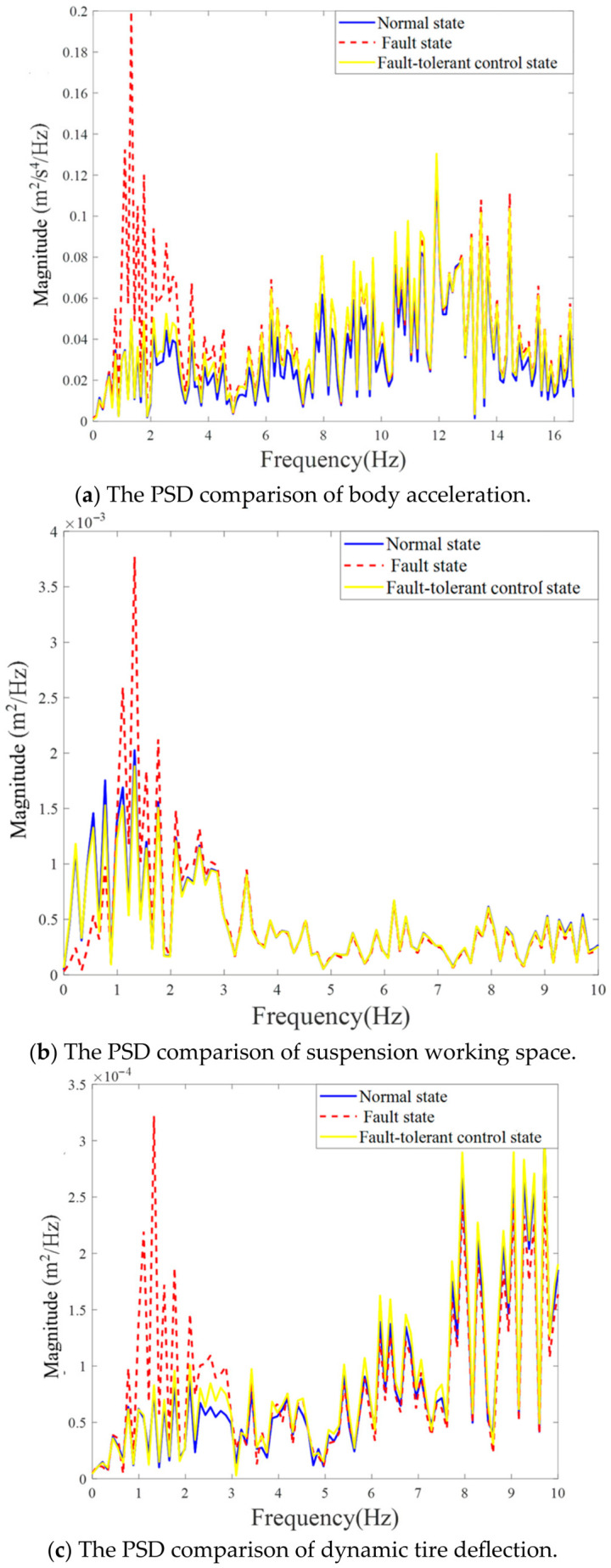
Comparison of power spectrum density for suspension performance index under different conditions.

Next, we analyze the effectiveness of this fault-tolerant control from a frequency domain perspective. By performing Fast Fourier Transform (FFT) on the body vertical acceleration, suspension working space, and dynamic tire deflection under three states, their corresponding power spectral densities (PSDs) are obtained, as shown in Figure 10.

As shown in Figure 10, under fault conditions within the 0~10 Hz frequency range, the fault-tolerant control state effectively suppresses the vehicle body’s vertical acceleration and suspension working space, bringing them close to normal, fault-free conditions. For dynamic tire deflection, a slight deterioration occurs above 4 Hz. This reflects the integrated application of control energy allocation, performance trade-offs, and multi-objective optimization. Within the vehicle body resonance frequency range of 1.0~1.5 Hz and the human body’s most sensitive frequency range of 4.0~10 Hz [40], the power spectral density is significantly reduced. This shows that the fault-tolerant control system has better vibration suppression performance in the main resonance frequency band. This mitigation lessens the damage caused by road impacts to both the vehicle body and vital human organs.

The comparison of time-domain responses and PSD curves in Figure 9 and Figure 10 clearly shows that the proposed fault-tolerant control effectively suppresses vibrations, bringing the system performance close to the normal state.

Therefore, this localized performance trade-off in non-core frequency bands represents a rational outcome under system safety constraints. It achieves global optimization of overall performance, thereby fully validating the engineering practicality and robustness of the designed fault-tolerant control strategy.

## 5. Conclusions

This paper proposes a fault-tolerant control framework for active suspension systems with actuator faults. An adaptive observer is designed to accurately estimate system states and actual fault forces, and an LQR-based controller is constructed to restore performance without reconfiguration. Simulation results show that the proposed method reduces the root mean square values of body vertical acceleration and suspension dynamic travel by 14.59% and 16.98%, respectively, effectively improving stability and ride comfort under faults. Future work will extend the method to multi-vehicle scenarios and complex road conditions.

## Figures and Tables

**Table 1:** Values of $\delta_{j}$ and $\beta_{j}$ in different modes.

Failure Mode	$\delta_{j}$	$\beta_{j}$
Normal	1	0
Gain fault	$0<\delta_{j}<1$	0
Constant deviation fault	1	$\beta_{j}$
Stuck fault	0	$\beta_{j}$

**Table 2:** Quarter-vehicle suspension parameters.

Symbol	Descriptions	Value and Unit
$m_{s}$	sprung mass	320 kg
$k_{s}$	suspension stiffness coefficients	18 KN/m
$c_{s}$	suspension damping coefficients	1 KN⋅s/m
$m_{u}$	unsprung mass	40 kg
$k_{t}$	tire stiffness coefficients	200 KN/m
$c_{t}$	tire damping coefficients	10 N⋅s/m

**Table 3:** The output root mean square (RMS) value of the time domain under grade B road conditions.

Indexes	N-S	F-S	F-T-C-S	F-T-E(%)
BA(m/s^2^)	0.3565	0.4834	0.4129	−14.59%
SWS(m)	0.0047	0.0053	0.0044	−16.98%
DTD(m)	0.0023	0.0020	0.0021	+5%

## Data Availability

Data are contained within the article.
